# P-1029. Victory in Battle, Defeat in War: Navigating Isavuconazole Implementation at an Argentinian Tertiary Centre

**DOI:** 10.1093/ofid/ofae631.1219

**Published:** 2025-01-29

**Authors:** Gustavo A Mendez, Jon Salmanton-Garcia, Carla Niveyro, Pedro A Villalba, Victoria Martin, Lucila Compañy-Kec, Maria C Tomasino, Vanesa M E Sosa, Oliver A Cornely, Haydee I Bernard

**Affiliations:** Hospital Escuela de Agudos Dr. Ramon Madariaga, Posadas, Misiones, Argentina; University Hospital Cologne, Cologne, Germany, Cologne, Nordrhein-Westfalen, Germany; Argentine society of infectology, Posadas, Misiones, Argentina; Argentine society of infectology, Posadas, Misiones, Argentina; Argentine society of infectology, Posadas, Misiones, Argentina; Infectious Diseases Department, Hospital Escuela de Agudos Dr. Ramón Madariaga, Posadas, Misiones, Argentina; Infectious Diseases Department, Hospital Escuela de Agudos Dr. Ramón Madariaga, Posadas, Misiones, Argentina; Laboratorio de Alta Complejidad de Misiones (LACMI), Posadas, Misiones, Argentina; University of Cologne, Faculty of Medicine and University Hospital Cologne, Cologne, Nordrhein-Westfalen, Germany; Infectious Diseases Department, Hospital Escuela de Agudos Dr. Ramón Madariaga, Posadas, Misiones, Argentina

## Abstract

**Background:**

Isavuconazole, a second-generation triazole, has emerged as a potent antifungal agent with broad spectrum activity against moulds, including aspergillosis and mucormycosis. Its favourable safety profile contributes to enhanced patient compliance and improved treatment outcomes.

**Methods:**

Data on patients receiving isavuconazole treatment from June 2019 to December 2023 were collected consecutively from an Argentinian tertiary care centre. A comprehensive range of variables was documented, including demographic details, underlying conditions, imaging findings, treatment specifics, and outcomes. Patients were categorized based on the EORTC/MSG criteria for invasive fungal infections.

**Results:**

In our study of 45 cases, we observed a prevalence of malignancies (60.0%), with 27 cases showing active malignancy. Imaging findings revealed common indicators of invasive fungal infection (IFI), notably pulmonary nodules (66.7%) and ground-glass opacity (62.2%). Breakthrough IFI occurred in 12 cases (26.7%). Isavuconazole was predominantly initiated as first-line treatment (68.9%), with a median duration of 59 days. Patients receiving first-line isavuconazole treatment were significantly older (51 vs. 32 years old, p=0.001) than those on subsequent lines. At 30 days, treatment response was observed in 20.0% of cases, with a mortality rate of 15.6%. Pulmonary nodules were significantly more frequent in patients on first-line isavuconazole treatment compared to subsequent lines (77.4% vs. 42.9%, p=0.039). Additionally, the presence of pulmonary nodules was significantly higher in patients with active IFI at day 30 compared to those with treatment response (84.0% vs. 44.4%, p=0.034).

**Conclusion:**

Our findings shed light on the complexities surrounding the use of isavuconazole in clinical practice and underscore the need for further research to optimize its application in managing invasive fungal infections. Mortality in isavuconazole-treated patients primarily stemmed from underlying disease progression rather than the fungal infection itself, emphasizing the necessity for a broad-spectrum

antifungal with fewer contraindications.

**Disclosures:**

**Oliver A. Cornely, Prof. Dr.**, Abbott: Honoraria|Abbvie: Advisor/Consultant|Abbvie: Honoraria|AiCuris: Advisor/Consultant|Akademie fur Infektionmedizin: Honoraria|Al-Jazeera Pharmaceuticals/Hikma: Honoraria|amedes: Honoraria|AstraZeneca: Honoraria|Basilea: Advisor/Consultant|Biocon: Advisor/Consultant|BMBF: Grant/Research Support|Boston Strategic Partners: Advisor/Consultant|CIdara: Advisor/Consultant|CIdara: Expert Testimony|CIdara: Grant/Research Support|CIdara: Participation on a DRC or DSMB|CoRe Consulting: Stocks/Bonds (Private Company)|Deutscher Arzteverlag: Honoraria|DZIF: Grant/Research Support|EasyRadiology: Stocks/Bonds (Private Company)|EU-DG RTD: Grant/Research Support|F2G: Grant/Research Support|Gilead: Advisor/Consultant|Gilead: Grant/Research Support|Gilead: Honoraria|Grupo Biotoscana/United Medical/Knight: Honoraria|GSK: Advisor/Consultant|GSK: Honoraria|IQVIA: Advisor/Consultant|IQVIA: Participation on a DRC or DSMB|Janssen: Advisor/Consultant|Janssen: Participation on a DRC or DSMB|Matinas: Advisor/Consultant|MedPace: Advisor/Consultant|MedPace: Grant/Research Support|MedPace: Participation on a DRC or DSMB|Medscape/WebMD: Honoraria|MedUpdate: Honoraria|Menarini: Advisor/Consultant|Moderna: Honoraria|Molecular Partners: Advisor/Consultant|MSD: Grant/Research Support|MSD: Honoraria|MSG-ERC: Advisor/Consultant|Mundipharma: Advisor/Consultant|Mundipharma: Grant/Research Support|Mundipharma: Honoraria|Noscendo: Honoraria|Noxxon: Advisor/Consultant|Octapharma: Advisor/Consultant|Octapharma: Grant/Research Support|Pardes: Advisor/Consultant|Partner Therapeutics: Advisor/Consultant|Patent: US18/562644|Paul-Martini-Stiftung: Honoraria|Pfizer: Advisor/Consultant|Pfizer: Grant/Research Support|Pfizer: Honoraria|PSI: Advisor/Consultant|PSI: Participation on a DRC or DSMB|Pulmocide: Participation on a DRC or DSMB|Sandoz: Honoraria|Scynexis: Advisor/Consultant|Scynexis: Grant/Research Support|Seqirus: Advisor/Consultant|Seqirus: Honoraria|Seres: Advisor/Consultant|Shionogi: Advisor/Consultant|Shionogi: Honoraria|streamedup!: Honoraria|The Prime Meridian Group: Advisor/Consultant|Touch Independent: Honoraria|Vitis: Honoraria

